# Child deaths caused by *Klebsiella pneumoniae* in sub-Saharan Africa and south Asia: a secondary analysis of Child Health and Mortality Prevention Surveillance (CHAMPS) data

**DOI:** 10.1016/S2666-5247(23)00290-2

**Published:** 2024-02

**Authors:** Jennifer R Verani, Dianna M Blau, Emily S Gurley, Victor Akelo, Nega Assefa, Vicky Baillie, Quique Bassat, Mussie Berhane, James Bunn, Anelsio C A Cossa, Shams El Arifeen, Revathi Gunturu, Martin Hale, Aggrey Igunza, Adama M Keita, Sartie Kenneh, Karen L Kotloff, Dickens Kowuor, Rita Mabunda, Zachary J Madewell, Shabir Madhi, Lola Madrid, Sana Mahtab, Judice Miguel, Florence V Murila, Ikechukwu U Ogbuanu, Julius Ojulong, Dickens Onyango, Joe O Oundo, J Anthony G Scott, Samba Sow, Milagritos Tapia, Cheick B Traore, Sithembiso Velaphi, Cynthia G Whitney, Inacio Mandomando, Robert F Breiman

**Affiliations:** a Center for Global Health, US Centers for Disease Control and Prevention, Nairobi, Kenya; b Center for Global Health, US Centers for Disease Control and Prevention, Atlanta, GA, USA; c Maternal and Child Health Division, International Center for Diarrhoeal Diseases Research (icddr,b), Dhaka, Bangladesh; d Department of Epidemiology, Johns Hopkins Bloomberg School of Public Health, Baltimore, MD, USA; e Center for Global Health, US Centers for Disease Control and Prevention Kenya, Kisumu, Kenya; f College of Health and Medical Sciences, Haramaya University, Harar, Ethiopia; g South African Medical Research Council Vaccines and Infectious Diseases Analytics Research Unit, University of the Witwatersrand, Johannesburg, South Africa; h ISGlobal - Hospital Clínic, Universitat de Barcelona, Barcelona, Spain; i Centro de Investigação em Saúde de Manhiça (CISM), Maputo, Mozambique; j Institució Catalana de Recerca I Estudis Avançats (ICREA), Barcelona, Spain; k Hospital Sant Joan de Déu, Barcelona, Spain; l Consorcio de Investigación Biomédica en Red de Epidemiología y Salud Pública (CIBERESP), Barcelona, Spain; m World Health Organization, Sierra Leone, Freetown, Sierra Leone; n Aga Khan University Hospital, Nairobi, Kenya; o National Health Laboratory Service, Department of Anatomical Pathology, School of Pathology, Faculty of Health Sciences, University of the Witwatersrand, Johannesburg, South Africa; p Kenya Medical Research Institute (KEMRI), Kisumu, Kenya; q Centre pour le Développement des Vaccins (CVD-Mali), Ministère de la Santé, Bamako, Mali; r Ministry of Health and Sanitation, Freetown, Sierra Leone; s Department of Pediatrics and Department of Medicine, Center for Vaccine Development and Global Health, University of Maryland School of Medicine, Baltimore, MD, USA; t Crown Agents, Freetown, Sierra Leone; u Department of Infectious Disease Epidemiology, London School of Hygiene & Tropical Medicine, London, UK; v University of Nairobi, Nairobi, Kenya; w ICAP – Columbia University, Makeni, Sierra Leone; x Kisumu County Department of Health, Kisumu, Kenya; y Department of Pathological Anatomy and Cytology, University Hospital of Point G, Bamako, Mali; z Department of Pediatrics, Chris Hani Baragwanath Academic Hospital, School of Clinical Medicine, Faculty of Health Sciences, University of the Witwatersrand, Johannesburg, South Africa; aa Department of Global Health, Rollins School of Public Health, Emory University, Atlanta, GA, USA; bb Instituto Nacional de Saúde (INS), Maputo, Mozambique; cc Infectious Diseases and Oncology Research Institute, University of the Witwatersrand, Johannesburg, South Africa

## Abstract

**Background:**

*Klebsiella pneumoniae* is an important cause of nosocomial and community-acquired pneumonia and sepsis in children, and antibiotic-resistant *K pneumoniae* is a growing public health threat. We aimed to characterise child mortality associated with this pathogen in seven high-mortality settings.

**Methods:**

We analysed Child Health and Mortality Prevention Surveillance (CHAMPS) data on the causes of deaths in children younger than 5 years and stillbirths in sites located in seven countries across sub-Saharan Africa (Ethiopia, Kenya, Mali, Mozambique, Sierra Leone, and South Africa) and south Asia (Bangladesh) from Dec 9, 2016, to Dec 31, 2021. CHAMPS sites conduct active surveillance for deaths in catchment populations and following reporting of an eligible death or stillbirth seek consent for minimally invasive tissue sampling followed by extensive aetiological testing (microbiological, molecular, and pathological); cases are reviewed by expert panels to assign immediate, intermediate, and underlying causes of death. We reported on susceptibility to antibiotics for which at least 30 isolates had been tested, and excluded data on antibiotics for which susceptibility testing is not recommended for *Klebsiella* spp due to lack of clinical activity (eg, penicillin and ampicillin).

**Findings:**

Among 2352 child deaths with cause of death assigned, 497 (21%, 95% CI 20–23) had *K pneumoniae* in the causal chain of death; 100 (20%, 17–24) had *K pneumoniae* as the underlying cause. The frequency of *K pneumoniae* in the causal chain was highest in children aged 1–11 months (30%, 95% CI 26–34; 144 of 485 deaths) and 12–23 months (28%, 22–34; 63 of 225 deaths); frequency by site ranged from 6% (95% CI 3–11; 11 of 184 deaths) in Bangladesh to 52% (44–61; 71 of 136 deaths) in Ethiopia. *K pneumoniae* was in the causal chain for 450 (22%, 95% CI 20–24) of 2023 deaths that occurred in health facilities and 47 (14%, 11–19) of 329 deaths in the community. The most common clinical syndromes among deaths with *K pneumoniae* in the causal chain were sepsis (44%, 95% CI 40–49; 221 of 2352 deaths), sepsis in conjunction with pneumonia (19%, 16–23; 94 of 2352 deaths), and pneumonia (16%, 13–20; 80 of 2352 deaths). Among *K pneumoniae* isolates tested, 121 (84%) of 144 were resistant to ceftriaxone and 80 (75%) of 106 to gentamicin.

**Interpretation:**

*K pneumoniae* substantially contributed to deaths in the first 2 years of life across multiple high-mortality settings, and resistance to antibiotics used for sepsis treatment was common. Improved strategies are needed to rapidly identify and appropriately treat children who might be infected with this pathogen. These data suggest a potential impact of developing and using effective *K pneumoniae* vaccines in reducing neonatal, infant, and child deaths globally.

**Funding:**

Bill & Melinda Gates Foundation.

## Introduction

*Klebsiella pneumoniae* are Gram-negative bacteria and an important nosocomial pathogen, particularly in immunocompromised adults and neonates.^1^, ^2^, ^3^ Studies have also highlighted the role of *K pneumoniae* as a cause of community-acquired infections, including neonatal sepsis.^3^^,^^4^ *K pneumoniae* commonly colonises humans, with prevalence ranging from 1% to 19% in the nasopharynx and 5% to 38% in the gastrointestinal tract.^1^^,^^5^ Neonates born to mothers with vaginal *K pneumoniae* colonisation can become colonised during the perinatal period.^6^ *K pneumoniae* can remain commensal, or infection can lead to illness with a wide range of clinical manifestations, including pneumonia, sepsis, genitourinary tract infections, intra-abdominal infections, meningitis, and soft tissue infections. Risk factors for progression from colonisation to disease are poorly understood,^5^ and geographical variation in the burden and clinical manifestations of community-acquired infections point to gaps in understanding of *K pneumoniae* epidemiology.^7^

**Research in context**

**Evidence before this study**

We searched PubMed for articles published from Jan 1, 2012, to March 31, 2022, using “Klebsiella” or “Klebsiella pneumoniae” as search terms, and focused on evidence of *K pneumoniae* disease in children and from low-income or middle-income settings. Numerous publications have reported on the role of *Klebsiella pneumoniae* as a cause of nosocomial pneumonia, particularly in immunocompromised individuals and neonates. *K pneumoniae* is described in published literature as an important cause of outbreaks in neonatal intensive care units. Community-acquired *K pneumoniae* infections are also described in the literature, although fewer studies have focused on this. Various publications reported on increasing antimicrobial resistance among *K pneumoniae* isolates, including multidrug-resistant and extremely drug-resistant strains. We also considered early findings from the Child Health and Mortality Prevention and Surveillance (CHAMPS) network, which aims to generate data on the causes of child deaths in areas with high child mortality in sub-Saharan Africa and south Asia. The first comprehensive publication of CHAMPS findings included data from five sites from December, 2016, to December, 2018, and reported that 53% of neonatal deaths and 90% of child deaths (ages 1–59 months) had at least one infectious condition contributing to the death; among deaths with a contributory pathogen identified, *K pneumoniae* was considered contributory to 31%, making it the second most frequently implicated pathogen in neonatal deaths and the most common among child deaths. However, beyond this description of the frequency with which *K pneumoniae* contributes to neonatal and child deaths, data on the role of *K pneumoniae* in child mortality are limited.

**Added value of this study**

This study used data from CHAMPS to provide more in-depth and current data on the contribution of *K pneumoniae* to child mortality, reporting on data from seven countries up to December, 2021. CHAMPS generates detailed and robust data on the causes of death among stillbirths and deaths among children aged 0–59 months. Sites conduct active surveillance for deaths among defined catchment populations and seek to enrol deaths and conduct minimally invasive tissue sampling within 24 h of the death. Samples undergo extensive aetiological testing (microbiological, molecular, and pathological). Expert panels review comprehensive data from medical record review, verbal autopsy, and laboratory results; panels assign immediate, intermediate, and underlying causes of death. In this study, we build on the previous CHAMPS report on the frequency of *K pneumoniae* contributing to child deaths by examining the role of *K pneumoniae* in deaths stratified by age group and site, describing *K pneumoniae*-associated clinical syndromes among fatal cases, and reporting on pathogens and underlying conditions that also contributed to deaths in which *K pneumoniae* played a role. We also present data on the antimicrobial susceptibility patterns of *K pneumoniae* isolates from child deaths with *K pneumoniae* in the causal chain of death.

**Implications of all the available evidence**

The findings of this study further highlight the importance of *K pneumoniae* as a cause of death among children aged 0–59 months in high-mortality settings across seven countries and provide evidence of both nosocomial and community-acquired *K pneumoniae* infections in these settings. Data on the age groups most commonly affected and with the highest burden of *K pneumoniae*-related deaths are crucial for informing prevention strategies, including potential development of vaccines against *K pneumoniae*. The data on antimicrobial susceptibility among *K pneumoniae* isolates from this study, together with existing evidence of increasing resistance among *K pneumoniae*, raise alarms about the ineffectiveness of commonly used antibiotics. Guidelines for empirical treatment of pneumonia and sepsis might require adaptation to optimally prevent deaths due to *K pneumoniae*, and infection prevention and control practices and antimicrobial stewardship efforts are crucial for curbing the spread of resistant *K pneumoniae* strains. The available evidence suggest that specific efforts aimed at reducing *K pneumoniae*-associated deaths will likely be necessary in order to meet Sustainable Development Goals for under-5 and neonatal mortality by 2030.

Increasing antimicrobial resistance among *K pneumoniae* isolates and the emergence of hypervirulent strains have compounded the threat from this pathogen.^8^ Multidrug-resistant and extremely drug-resistant strains are increasingly common, greatly limiting the available antibiotic options for treating *K pneumoniae* infections.^9^^,^^10^ Studies have reported that carbapenem-resistant *K pneumoniae* strains are associated with increased case fatality.^11^^,^^12^

The role of *K pneumoniae* in child death is not well understood, particularly among children in resource-poor settings. The Child Health and Mortality Prevention Surveillance (CHAMPS) network tracks the burden and causes of deaths in children younger than 5 years and stillbirths in seven sites across sub-Saharan Africa and south Asia.^13^ Initial findings (published in 2020) from the first five sites found *K pneumoniae* to be a common contributory pathogen among child deaths with an infectious cause.^14^ We aim to characterise key clinical and epidemiological features of *K pneumoniae*-associated child mortality and stillbirths in CHAMPS site settings.

## Methods

### Study design and population

We conducted a secondary analysis of CHAMPS data. The CHAMPS methods for identifying deaths and determining the causes among children younger than 5 years and stillbirths have been described elsewhere^13^^,^^14^ and are briefly summarised here. This analysis included data from CHAMPS surveillance sites located in seven countries: Baliakandi, Bangladesh; Harar, Haramaya, and Kersa, Ethiopia; Siaya and Kisumu, Kenya; Bamako, Mali; Quelimane and Manhiça, Mozambique; Bombali, Sierra Leone; and Soweto, South Africa. All sites are high-mortality settings, and the network includes a mix of rural and urban sites.^15^ Stillbirths and deaths among children younger than 5 years who reside in catchment areas are promptly reported from health facilities and via networks of community reporters. Upon receiving notification of an eligible stillbirth or death, CHAMPS staff rapidly approach family members to screen for eligibility and seek written informed consent for collection of data and specimens.

Ethics committees overseeing investigators at each site and at Emory University approved overall and site-specific protocols (Emory institutional review board number 00091706). Protocols are available on the CHAMPS website.

### Procedures

Data collection (verbal autopsy using the WHO 2016 instrument, clinical record abstraction, anthropometric measurements, and photographs) was performed using standardised procedures by trained staff. Deaths for which CHAMPS staff were notified within 36 h (or ≤72 h if post-mortem refrigeration was used) were eligible for minimally invasive tissue sampling (MITS). MITS specimens were collected using biopsy needles under sterile conditions. Tissues sampled included the lungs, heart, brain, liver, bone marrow, and placenta (for stillbirths). In addition, samples of blood, cerebrospinal fluid (CSF), stool, and nasopharyngeal secretions were collected.

Laboratories at each site performed culture of blood and CSF using standard microbiological procedures. For sites with automated culture systems, identification of *K pneumoniae* was performed by automated systems (Vitek 2 [bioMerieux, Hazelwood, MO, USA], BD Phoenix [BD, Franklin Lakes, NJ, USA] or MicroScan [Beckman Coulter, Brea, CA, USA]). For sites without access to automated systems, specimens with growth were inoculated onto blood and MacConkey agars and incubated for 18–24 h; identification of pure colonies was performed by biochemical reactions and colony morphology, and further confirmation was performed by *K pneumoniae*-specific PCR.

Antimicrobial susceptibility testing of *K pneumoniae* isolates was not a standardised CHAMPS process; however, sites used either automated systems (Vitek 2, BD Phoenix, or MicroScan) or the Kirby–Bauer disk diffusion method. Isolates were classified as susceptible, intermediate, or resistant according to Clinical and Laboratory Standards Institute guidance.^16^ Specific antibiotics tested and proportion of *K pneumoniae* isolates that underwent susceptibility testing varied by site; we reported on antibiotics for which at least 30 isolates had been tested, and excluded data on antibiotics for which susceptibility testing is not recommended for *Klebsiella* spp due to lack of clinical activity (eg, penicillin and ampicillin).^16^ Site laboratories also tested post-mortem blood samples for HIV and prepared and examined thin and thick smears for malaria using microscopy. Five custom TaqMan Array Cards (TAC, ThermoFisher Scientific, Waltham, MA, USA) were used for molecular detection of 116 pathogens from lung tissue, blood, CSF, rectal swabs, and nasopharyngeal swabs.^17^ *K pneumoniae* targets were included on the TAC panels for lung tissue, blood, CSF, and nasopharyngeal swabs. Histopathological analyses of all tissues using routine stains was performed at site pathology laboratories and at the US Centers for Disease Control and Prevention (CDC) in Atlanta, GA, USA. Additionally, when indicated by TAC, blood culture, or histopathology findings, additional testing including Gram stain and immunohistochemistry (IHC) was done at the CDC pathology laboratories. The select Gram-negative bacterial IHC assay uses an antibody raised against *K pneumoniae* but is known to be broadly cross-reactive with other Gram-negative bacteria of relevance to CHAMPS cases, including but not limited to *Escherichia coli*, *Pseudomonas* spp, and *Haemophilus influenzae*.

Determination of cause of death (DeCoDe) panels at each site included paediatricians, obstetricians, epidemiologists, pathologists, and microbiologists.^18^ The panels reviewed all available data for each case and assigned the chain of events leading to death using the tenth revision of the International Classification of Diseases (ICD-10) for deaths among children aged older than 28 days, and ICD-perinatal mortality (ICD-PM) for deaths in the perinatal or neonatal period. For deaths with more than one cause, the panels assigned (1) the immediate or most proximal cause, (2) antecedent or comorbid causes that contributed directly to the chain, and (3) the underlying cause that initiated the chain of events that led to the death. We defined the causal chain for death to be conditions included in any of the three categories. For any pathogen detected, the DeCoDe standards provided levels of certainty for the finding, by linking microbiological with histopathological and clinical data.^19^ The DeCoDe process was standardised across sites using network-wide diagnosis standards and through training.^18^

### Outcomes and statistical analysis

We conducted a descriptive analysis of deaths with *K pneumoniae* in the causal chain from Dec 9, 2016, to Dec 31, 2021. Because *K pneumoniae* was infrequently deemed to be in the causal chain of death for stillbirths, we present only the overall proportion of stillbirths with *K pneumoniae* in the causal chain; subsequent analyses were restricted to child deaths only. Deaths were classified as community or health facility deaths based on the location of the child at the time of death; facility deaths were stratified in those occurring 48 h or less after admission, for which the source of *K pneumoniae* was more likely from the community, and those occurring more than 48 h after admission, for which the source of infection was more likely nosocomial. We used a multivariable logistic regression model to examine the association of age, location of death, and site with having *K pneumoniae* in the causal chain (*vs* death without *K pneumoniae* in the causal chain). All statistical analyses were conducted using R software, version 4.1.2.

### Role of the funding source

The study sponsor was involved in discussions about design of the CHAMPS network; the sponsor had no role in collection, analysis or interpretation of data, writing of this manuscript, or the decision to publish.

## Results

From Dec 9, 2016, to Dec 31, 2021, 3030 stillbirths were reported; consent was provided for 1528 (50%), MITS was performed on 1489 (97%) of 1528, and DeCoDe was completed for 1297 (87%) of 1489 (appendix p 2). *K pneumoniae* was found to be in the causal chain for five (<1%) stillbirths (table 1), including four (2%, 95% CI 1–5) in South Africa and one (1%, 0–4) in Sierra Leone. Among 6320 child deaths reported, consent was provided for 2797 (44%), MITS was performed on 2763 (99%) of 2797, and DeCoDe was completed for 2352 (85%) of 2763 (appendix p 3). *K pneumoniae* was in the causal chain for 497 (21%, 95% CI 20–23) of 2352 child deaths, including 197 (19%, 17–22) of 1030 among females and 300 (23%, 21–25) of 1319 among males; for three deaths, the sex was indeterminate, none of which had *K pneumoniae* in the causal chain. Among child deaths with *K pneumoniae* in the causal chain, *K pneumoniae* was an underlying cause of 100 (20%, 95% CI 17–24), including 25 (5%, 3–7) for which *K pneumoniae* was the only cause listed (ie, *K pneumoniae* both immediate and underlying cause). *K pneumoniae* was considered part of the causal chain but with another cause as underlying in 397 (80%, 95% CI 76–83) child deaths, including as a comorbid condition in 76 (15%, 12–19), an immediate cause of death in 186 (37%, 33–42), and both a comorbid condition and an immediate cause of death in 135 (27%, 23–31). The median time from death to MITS for all deaths was 12 h (IQR 4–21) and for deaths with *K pneumoniae* in the causal chain was 13 h (4–22).

**Table 1:** Frequency of deaths with *Klebsiella pneumoniae* in the causal chain, associated clinical syndromes, and laboratory evidence of *K pneumoniae*, by age group

	Stillbirths		Neonates		1–11 months		12–23 months		24–59 months		Child deaths at all ages∗	
	n	% (95% CI)	n	% (95% CI)	n	% (95% CI)	n	% (95% CI)	n	% (95% CI)	n	% (95% CI)
**Deaths with MITS with COD determination**												
Overall	1297	··	1424	··	485	··	225	··	218	··	2352	··
**Deaths with *K pneumoniae* in causal chain of death**												
Overall	5	0% (0–1)	257	18% (16–20)	144	30% (26–34)	63	28% (22–34)	33	15% (11–21)	497	21% (20–23)
*K pneumoniae* underlying COD†	4	80% (30–99)	62	24% (19–30)	28	19% (14–27)	8	13% (6–24)	2	6% (1–22)	100	20% (17–24)
*K pneumoniae* only cause	2	40% (7–83)	21	8% (5–12)	4	3% (1–7)	0	0% (0–7)	0	0% (0–13)	25	5% (3–7)
*K pneumoniae* underlying only	4	80% (30–99)	41	16% (12–21)	17	12% (7–18)	6	10% (4–20)	2	6% (1–22)	66	13% (10–17)
*K pneumoniae* underlying and immediate COD but not comorbid	0	0% (0–54)	15	6% (3–10)	9	6% (3–12)	2	3% (1–12)	0	0% (0–13)	26	5% (4–8)
*K pneumoniae* underlying and comorbid but not immediate COD	0	0% (0–54)	3	1% (0–4)	1	1% (0–4)	0	0% (0–7)	0	0% (0–13)	4	1% (0–2)
*K pneumoniae* underlying and immediate COD and comorbid	0	0% (0–54)	3	1% (0–4)	1	1% (0–4)	0	0% (0–7)	0	0% (0–13)	4	1% (0–2)
*K pneumoniae* in causal chain but not underlying COD	1	20% (1–70)	195	76% (70–81)	116	81% (73–86)	55	87% (76–94)	31	94% (78–99)	397	80% (76–83)
*K pneumoniae* comorbid condition only	1	20% (1–70)	31	12% (8–17)	23	16% (11–23)	13	21% (12–33)	9	27% (14–46)	76	15% (12–19)
*K pneumoniae* immediate COD only	0	0% (0–54)	84	33% (27–39)	55	38% (30–47)	31	49% (37–62)	16	48% (31–66)	186	37% (33–42)
*K pneumoniae* immediate and comorbid COD	0	0% (0–54)	80	31% (26–37)	38	26% (20–35)	11	17% (9–30)	6	18% (8–36)	135	27% (23–31)
***K pneumoniae*-associated clinical syndrome in deaths with *K pneumoniae* in causal chain**												
Sepsis	3	60% (17–93)	125	49% (42–55)	54	38% (30–46)	29	46% (34–59)	13	39% (23–58)	221	44% (40–49)
Pneumonia	0	0% (0–54)	19	7% (5–11)	32	22% (16–30)	17	27% (17–40)	12	36% (21–55)	80	16% (13–20)
Sepsis and pneumonia	0	0% (0–54)	42	16% (12–22)	36	25% (18–33)	11	17% (9–30)	5	15% (6–33)	94	19% (16–23)
Meningitis	1	20% (1–70)	3	1% (0–4)	5	3% (1–8)	0	0% (0–7)	0	0% (0–13)	8	2% (1–3)
Sepsis and meningitis	0	0% (0–54)	21	8% (5–12)	6	4% (2–9)	1	2% (0–10)	0	0% (0–13)	28	6% (4–8)
Pneumonia and meningitis	0	0% (0–54)	4	2% (0–4)	1	1% (0–4)	0	0% (0–7)	0	0% (0–13)	5	1% (0–2)
Sepsis, pneumonia, and meningitis	0	0% (0–54)	25	10% (7–14)	6	4% (2–9)	3	5% (1–14)	2	6% (1–22)	36	7% (5–10)
Sepsis and other clinical syndrome‡	0	0% (0–54)	9	4% (2–7)	1	1% (0–4)	0	0% (0–7)	0	0% (0–13)	10	2% (1–4)
Sepsis, meningitis, and other clinical syndrome	0	0% (0–54)	2	1% (0–3)	0	0% (0–3)	0	0% (0–7)	0	0% (0–13)	2	0% (0–2)
Other clinical syndrome	1	20% (1–70)	6	2% (1–5)	3	2% (1–6)	1	2% (0–10)	1	3% (0–18)	11	2% (1–4)
**Laboratory evidence of *K pneumoniae* among deaths with *K pneumoniae* in causal chain**												
Culture§, TAC, and IHC	0	0% (0–54)	46	18% (14–23)	24	17% (11–24)	10	16% (8–28)	7	21% (10–39)	87	18% (14–21)
Culture and TAC	2	40% (7–83)	135	53% (46–59)	69	48% (40–56)	30	48% (35–60)	12	36% (21–55)	248	50% (45–54)
TAC and IHC	0	0% (0–54)	15	6% (3–10)	11	8% (4–14)	11	17% (9–30)	4	12% (4–29)	41	8% (6–11)
Culture only	2	40% (7–83)	13	5% (3–9)	5	3% (1–8)	0	0% (0–7)	1	3% (0–18)	21	4% (3–6)
TAC only	1	20% (1–70)	45	18% (13–23)	33	23% (17–31)	12	19% (11–31)	8	24% (12–43)	99	20% (17–24)
IHC only	0	0% (0–54)	1	0% (0–2)	0	0% (0–3)	0	0% (0–7)	0	0% (0–13)	1	0% (0–1)
No post-mortem laboratory evidence of *K pneumoniae*	0	0% (0–54)	2	1% (0–3)	2	1% (0–5)	0	0% (0–7)	1	3% (0–18)	5	1% (0–2)

MITS=minimally invasive tissue sampling. COD=cause of death. TAC=Taqman array card, performed on blood, cerebrospinal fluid, and lung tissue. IHC=immunohistochemistry, performed on lung, liver, heart, and brain; not performed in all cases.

∗ Excludes stillbirths.

† “*K pneumoniae* only cause” refers to deaths with *K pneumoniae* as the underlying COD with no other cause noted, while “*K pneumoniae* underlying only” refers to deaths with *K pneumoniae* as the underlying COD (but not immediate COD or comorbid condition), with or without other causes in the causal chain; these two categories are not mutually exclusive.

‡ Congenital infection, other respiratory disease, and other infections.

§ Culture performed on blood and cerebrospinal fluid.

**Figure 1:**
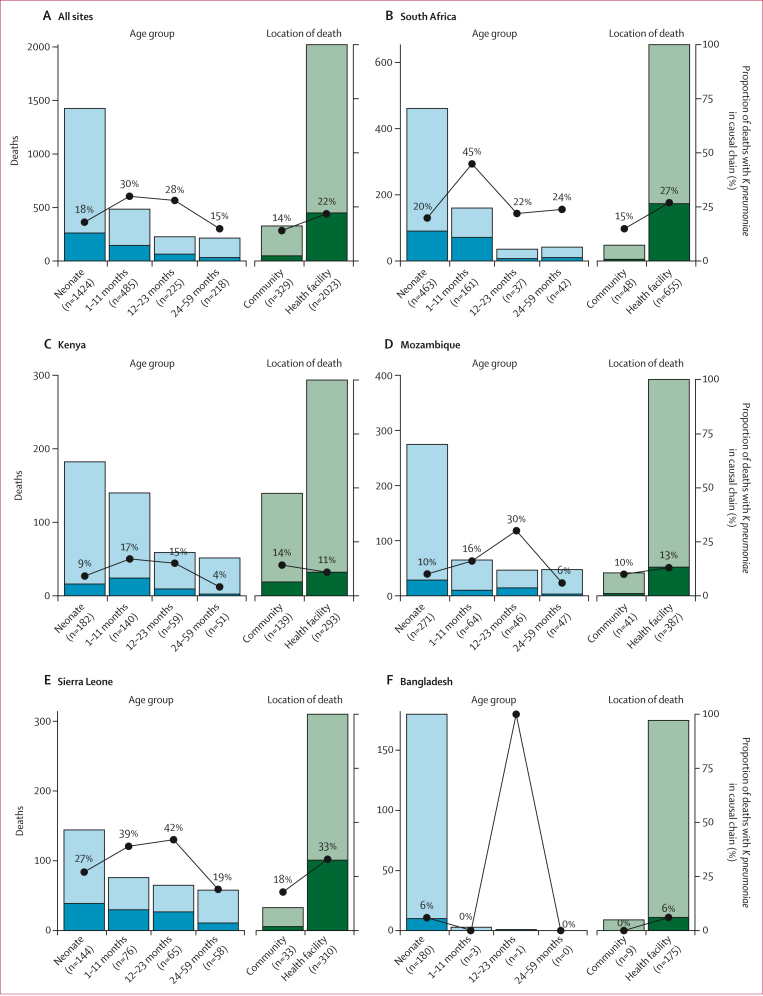

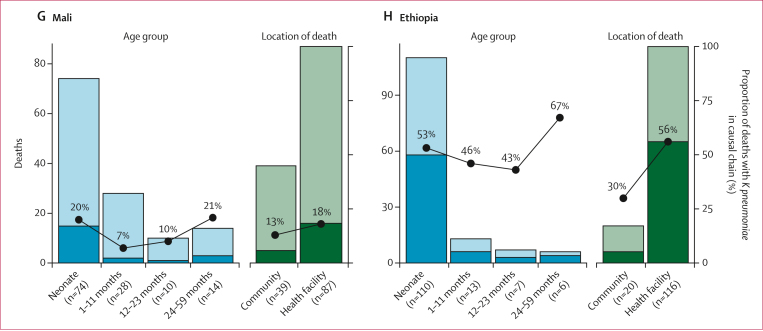
Deaths with *Klebsiella pneumoniae* in the causal chain by age group, location of death, and site The height of each bar reflects total deaths in that category included in the analysis; the darker portion of the bar represents the subset of those deaths with *K pneumoniae* in the causal chain.

Among all child deaths, *K pneumoniae* was identified in the causal chain more commonly among deaths in children aged 1–11 months (30%, 95% CI 26–34; 144 of 485 deaths) and 12–23 months (28%, 22–34, 63 of 225 deaths) than among deaths in other age groups. By site, the proportion of deaths with *K pneumoniae* in the causal chain ranged from a low of 6% (95% CI 3–11; 11 of 184 deaths) in Bangladesh to a high of 52% (44–61; 71 of 136 deaths) in Ethiopia (table 2). For neonatal deaths, *K pneumoniae* was in the causal chain for 18% (95% CI 16–20; 257 of 1424 deaths) overall; however, the proportion ranged across sites, from 9% (95% CI 5–14; 16 of 182 deaths) in Kenya to 53% (43–62; 58 of 110 deaths) in Ethiopia (figure 1).

**Table 2:** Frequency of child deaths with *Klebsiella pneumoniae* in the causal chain, associated clinical syndromes, and laboratory evidence of *K pneumoniae*, by site

	South Africa		Kenya		Mozambique		Sierra Leone		Bangladesh		Mali		Ethiopia	
	n	% (95% CI)	n	% (95% CI)	n	% (95% CI)	n	% (95% CI)	n	% (95% CI)	n	% (95% CI)	n	% (95% CI)
**Deaths with MITS with COD determination**														
Overall	703	··	432	··	428	··	343	··	184	··	126	··	136	··
**Deaths with *K pneumoniae* in causal chain of death**														
Overall	181	26% (23–29)	51	12% (915)	55	13% (10–16)	107	31% (26–36)	11	6% (3–11)	21	17% (11–25)	71	52% (44–61)
*K pneumoniae* underlying COD∗	14	8% (4–13)	18	35% (23–50)	20	36% (24–50)	34	32% (23–42)	4	36% (12–68)	2	10% (2–32)	8	11% (5–22)
*K pneumoniae* only cause	2	1% (0–4)	5	10% (4–22)	11	20% (11–33)	4	4% (1–10)	1	9% (0–43)	2	10% (2–32)	0	0% (0–6)
*K pneumoniae* underlying only	11	6% (3–11)	13	25% (15–40)	15	27% (17–41)	20	19% (12–28)	3	27% (7–61)	2	10% (2–32)	2	3% (0–11)
*K pneumoniae* underlying and immediate COD but not comorbid	1	1% (0–4)	5	10% (4–22)	5	9% (3–21)	13	12% (7–20)	1	9% (0–43)	0	0% (0–19)	1	1% (0–9)
*K pneumoniae* underlying and comorbid but not immediate COD	1	1% (0–4)	0	0% (0–9)	0	0% (0–8)	1	1% (0–6)	0	0% (0–32)	0	0% (0–19)	2	3% (0–11)
*K pneumoniae* underlying and immediate COD and comorbid	1	1% (0–4)	0	0% (0–9)	0	0% (0–8)	0	0% (0–4)	0	0% (0–32)	0	0% (0–19)	3	4% (1–13)
*K pneumoniae* in causal chain but not underlying COD	167	92% (87–96)	33	65% (50–77)	35	64% (50–76)	73	68% (58–77)	7	64% (32–88)	19	90% (68–98)	63	89% (78–95)
*K pneumoniae* comorbid condition only	37	20% (15–27)	6	12% (5–25)	2	4% (1–14)	17	16% (10–25)	0	0% (0–32)	5	24% (9–48)	9	13% (6–23)
*K pneumoniae* immediate COD only	64	35% (29–43)	21	41% (28–56)	28	51% (37–64)	46	43% (34–53)	5	45% (18–75)	5	24% (9–48)	17	24% (15–36)
*K pneumoniae* immediate and comorbid COD	66	36% (30–44)	6	12% (5–25)	5	9% (3–21)	10	9% (5–17)	2	18% (3–52)	9	43% (23–66)	37	52% (40–64)
***K pneumoniae*-associated clinical syndrome in deaths with *K pneumoniae* in causal chain**														
Sepsis	66	36% (30–44)	27	53% (39–67)	27	49% (36–63)	64	60% (50–69)	8	73% (39–93)	9	43% (23–66)	20	28% (18–40)
Pneumonia	32	18% (13–24)	16	31% (20–46)	14	25% (15–39)	12	11% (6–19)	0	0% (0–32)	1	5% (0–26)	5	7% (3–16)
Sepsis and pneumonia	44	24% (18–31)	6	12% (5–25)	8	15% (7–27)	15	14% (8–22)	0	0% (0–32)	9	43% (23–66)	12	17% (9–28)
Meningitis	4	2% (1–6)	1	2% (0–12)	0	0% (0–8)	3	3% (1–9)	0	0% (0–32)	0	0% (0–19)	0	0% (0–6)
Sepsis and meningitis	7	4% (2–8)	0	0% (0–9)	0	0% (0–8)	8	7% (4–15)	2	18% (3–52)	0	0% (0–19)	11	15% (8–26)
Pneumonia and meningitis	4	2% (1–6)	1	2% (0–12)	0	0% (0–8)	0	0% (0–4)	0	0% (0–32)	0	0% (0–19)	0	0% (0–6)
Sepsis, pneumonia, and meningitis	20	11% (7–17)	0	0% (0–9)	0	0% (0–8)	0	0% (0–4)	0	0% (0–32)	2	10% (2–32)	14	20% (12–31)
Sepsis and other clinical syndrome†	1	1% (0–4)	0	0% (0–9)	1	2% (0–11)	1	1% (0–6)	1	9% (0–43)	0	0% (0–19)	6	8% (3–18)
Sepsis, meningitis, and other clinical syndrome	0	0% (0–3)	0	0% (0–9)	0	0% (0–8)	0	0% (0–4)	0	0% (0–32)	0	0% (0–19)	2	3% (0–11)
Other clinical syndrome	3	2% (0–5)	0	0% (0–9)	4	7% (2–18)	3	3% (1–9)	0	0% (0–32)	0	0% (0–19)	1	1% (0–9)
**Laboratory evidence of *K pneumoniae* among deaths with *K pneumoniae* in causal chain**														
Culture‡, TAC, and IHC	30	17% (12–23)	10	20% (10–34)	14	25% (15–39)	17	16% (10–25)	3	27% (7–61)	5	24% (9–48)	8	11% (5–22)
Culture and TAC	87	48% (41–56)	14	27% (16–42)	26	47% (34–61)	60	56% (46–66)	5	45% (18–75)	12	57% (34–77)	42	59% (47–70)
TAC and IHC	9	5% (2–10)	13	25% (15–40)	5	9% (3–21)	7	7% (3–13)	1	9% (0–43)	3	14% (4–37)	3	4% (1–13)
Culture only	12	7% (4–12)	0	0% (0–9)	0	0% (0–8)	3	3% (1–9)	1	9% (0–43)	0	0% (0–19)	3	4% (1–13)
TAC only	38	21% (15–28)	13	25% (15–40)	10	18% (10–31)	20	19% (12–28)	1	9% (0–43)	1	5% (0–26)	15	21% (13–33)
IHC only	0	0% (0–3)	1	2% (0–12)	0	0% (0–8)	0	0% (0–4)	0	0% (0–32)	0	0% (0–19)	0	0% (0–6)
No post-mortem laboratory evidence of *K pneumoniae*	5	3% (1–7)	0	0% (0–9)	0	0% (0–8)	0	0% (0–4)	0	0% (0–32)	0	0% (0–19)	0	0% (0–6)

MITS=minimally invasive tissue sampling. COD=cause of death. TAC=Taqman array card, performed on blood, cerebrospinal fluid, and lung tissue. IHC=immunohistochemistry, performed on lung, liver, heart, and brain; not performed in all cases.

∗ "*K pneumoniae* only cause" refers to deaths with *K pneumoniae* as the underlying COD with no other cause noted, while "*K pneumoniae* underlying only" refers to deaths with *K pneumoniae* as the underlying COD (but not immediate COD or comorbid condition), with or without other causes in the causal chain; these two categories are not mutually exclusive.

† Congenital infection, other respiratory disease, and other infections.

‡ Culture performed on blood and cerebrospinal fluid.

*K pneumoniae* was in the causal chain for 450 (22%, 95% CI 20–24) of 2023 deaths that occurred in health facilities and 47 (14%, 11–19) of 329 that occurred in the community. In Kenya, the site with the largest number of community deaths undergoing MITS, the proportion of deaths with *K pneumoniae* in the causal chain was higher in community (14%, 95% CI 9–21; 19 of 139 deaths) than among health facility deaths (11%, 8–15; 32 of 293 deaths). Among 47 deaths that occurred in the community with *K pneumoniae* in the causal chain across all sites, 30 had information on care-seeking before death and 19 (63%, 44–79) of those had been admitted to hospital during the course of illness; for the remaining 11 (37%, 21–56), no substantial exposure to health-care facilities was noted. Among the 450 health facility deaths with *K pneumoniae* in the causal chain from all sites, the median duration between hospitalisation and death was 5 days (IQR 2–11); 262 (58%, 95% CI 53–63) died more than 48 h after arrival to the facility.

Results of a multivariable model comparing deaths with and without *K pneumoniae* in the causal chain with regard to age, site, and location of death were consistent with descriptive analyses; age 1–11 months, age 12–23 months, Ethiopia site, and death occurring in hospital more than 48 h after admission all had adjusted odds ratios greater than 1 (appendix p 7).

The most common clinical syndromes among the 497 deaths with *K pneumoniae* in the causal chain were sepsis (44%, 95% CI 40–49), sepsis in conjunction with pneumonia (19%, 16–23), and pneumonia (16%, 13–20); 2% (1–3) had meningitis without pneumonia or sepsis (table 1). The predominance of sepsis and pneumonia was consistent across sites (table 2). Among children with *K pneumoniae* sepsis episodes contributing to deaths, 195 (50%, 95% CI 45–55) of 391 had multipathogen sepsis, and among *K pneumoniae* pneumonia episodes contributing to deaths, 143 (67%, 60–73) of 215 were multipathogen pneumonia. Other pathogens most commonly implicated together with *K pneumoniae* in multipathogen sepsis or pneumonia were *Acinetobacter baumannii* (n=87; 32%, 95% CI 26–38), *E coli* (n=57; 21%, 16–26), *Streptococcus pneumoniae* (n=45; 16%, 12–22), and *Pseudomonas aeruginosa* (n=30; 11%, 8–15; appendix p 4). The distribution of other pathogens involved in multipathogen sepsis or pneumonia varied by location and timing of death; *A baumannii* was more common in deaths that occurred more than 48 h after admission (50%, 95% CI 42–58; 76 of 153 deaths), whereas *S pneumoniae* and *E coli* were more common among deaths that occurred in the community (45%, 30–64, 13 of 29 deaths; and 31%, 16–51, nine of 29 deaths, respectively) and within 48 h of admission to hospital (29%, 20–39, 26 of 91 deaths; and 26%, 18–37, 24 of 91 deaths, respectively). Meningitis was multipathogen in 27 (34%, 95% CI 24–46) of 79 cases.

**Figure 2:**
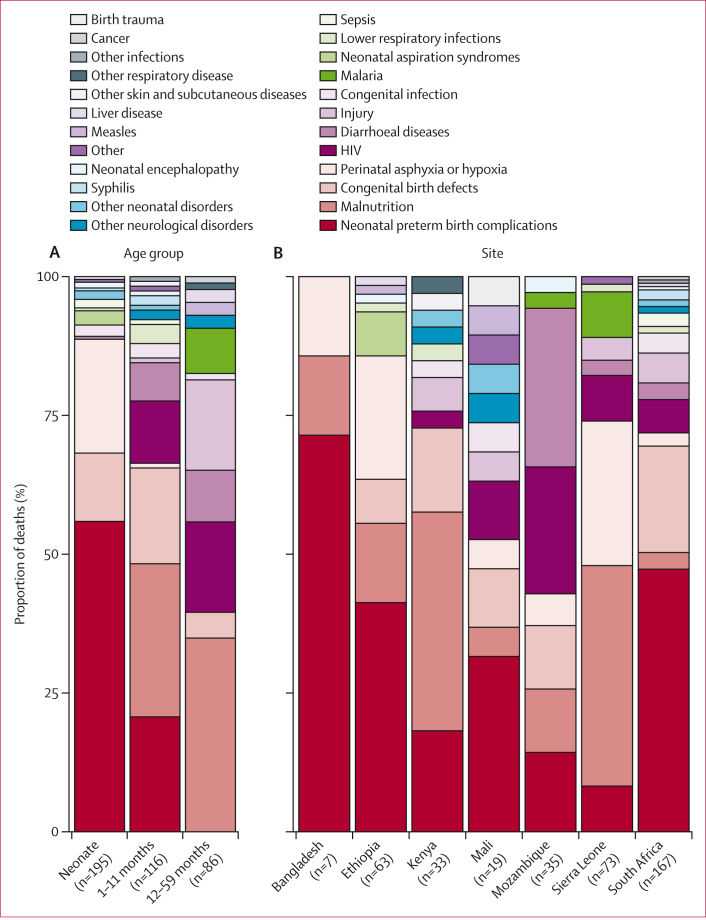
Underlying causes of death among deaths with *Klebsiella pneumoniae* as immediate cause of death or comorbid condition among all sites, by age group (A) and site (B)

Among deaths in which *K pneumoniae* was either an immediate cause of death or a comorbid condition, the most common underlying causes varied by age group (figure 2A; appendix p 5). Among neonates with *K pneumoniae* as an immediate or comorbid condition, the most common underlying causes were preterm birth complications (56%, 95% CI 49–63), perinatal asphyxia or hypoxia (21%, 15–27), and congenital birth defects (12%, 8–18). Among infants aged 1–11 months, malnutrition (28%, 95% CI 20–37), preterm birth complications (21%, 14–29), congenital birth defects (17%, 11–26), and HIV (11%, 6–19) were the most frequent underlying causes. Among children aged 12–59 months, malnutrition (35%, 95% CI 25–46) was the most common underlying cause, followed by injury (16%, 10–26) and HIV (16%, 10–26). Variations in underlying causes of death across sites (figure 2B; appendix p 6) reflected differences in age distribution.

Among the 2352 child deaths that underwent MITS and had DeCoDe results during the study period, *K pneumoniae* was detected by TAC in at least one specimen of 1297 (55%, 95% CI 53–57); 36% (34–39) of those with *K pneumoniae* detected by TAC were found to have *K pneumoniae* in the causal pathway, thus 64% (61–66) of these detections were judged to be incidental and not related to death. Among 349 deaths in which *K pneumoniae* was detected solely through TAC testing of nasopharyngeal swabs, 22 (6%, 95% CI 4–10) had *K pneumoniae* in the causal chain, 21 of which were deaths that occurred in health facilities. Among 948 deaths in which *K pneumoniae* was detected by TAC on either blood, lung, or CSF, 450 (47%, 95% CI 44–51) had *K pneumoniae* in the causal chain. Among all deaths with *K pneumoniae* in the causal pathway, 248 (50%, 95% CI 45–54) had evidence of *K pneumoniae* by culture (blood or CSF, or both) and by TAC (on blood, CSF, or lung tissue, or a combination of these samples), and 87 (18%, 14–21) had evidence of *K pneumoniae* by culture (blood or CSF, or both), by TAC (on blood, CSF, lung tissue, or a combination), and by IHC (lung, liver, heart, or brain, or a combination); 99 (20%, 17–24) had *K pneumoniae* detected by TAC only (table 1). Five deaths had no post-mortem laboratory evidence of *K pneumoniae* but had *K pneumoniae* isolated from antemortem blood cultures.

**Figure 3:**
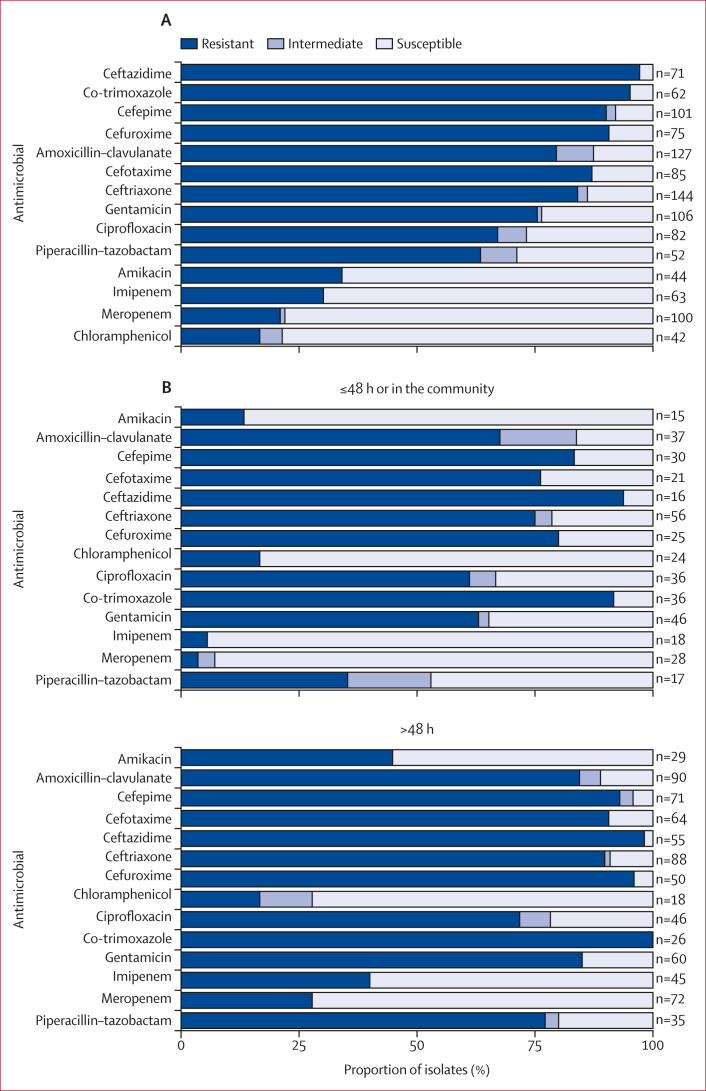
Antimicrobial susceptibility of *Klebsiella pneumoniae* isolates from deaths with *K pneumoniae* in the causal chain Overall (A) and stratified by deaths occurring in the community or within 48 h of admission and those occurring more than 48 h after admission (B).

Of 497 child deaths with *K pneumoniae* in the causal chain across all sites, 157 (32%, 95% CI 28–36) had *K pneumoniae* isolates that underwent antimicrobial susceptibility testing for relevant antibiotics. The number of isolates tested for each antibiotic ranged from 42 (chloramphenicol) to 144 (ceftriaxone; figure 3A). Resistance to ceftriaxone and gentamicin was observed in 121 (84%, 95% CI 77–89) of 144 and 80 (75%, 66–83) of 106 isolates tested, respectively. The frequency of resistance to imipenem and meropenem was 30% (95% CI 20–43; 19 of 63 isolates) and 21% (14–31; 21 of 100 isolates), respectively. Resistance to most antibiotics tested was more common among deaths that occurred more than 48 h after admission (figure 3B).

## Discussion

This analysis of comprehensive child mortality data from seven countries highlights the importance of *K pneumoniae* as a cause of death among children under 5 years in resource-poor settings. *K pneumoniae* was implicated in more than one in four deaths among children aged 1–23 months. While the proportion of neonatal deaths with *K pneumoniae* in the causal chain was lower (18%) than in older age groups, the high mortality rate among neonates, especially in sub-Saharan Africa and Asia^20^ yields a substantial global burden of neonatal deaths in which *K pneumoniae* appears to play a causal role. Our results also illustrate the complexity of factors contributing to child deaths;^21^ *K pneumoniae* was frequently identified in conjunction with other pathogens and in children with prematurity, birth complications, HIV, and malnutrition. If Sustainable Development Goals for under-5 and neonatal mortality are to be met by 2030,^22^ specific efforts aimed at reducing *K pneumoniae*-associated deaths, including multifaceted interventions addressing multiple interacting conditions contributing to mortality will likely be necessary.^21^

We found *K pneumoniae* to play an important role in deaths occurring both in the community and in health facilities. The proportion of community deaths and health facility deaths with *K pneumoniae* in the causal chain varied across sites; however, interpretation is limited by small numbers of community deaths at several sites. Of note, the location of death is not necessarily reflective of where the exposure to *K pneumoniae* occurred. For many of the in-hospital deaths with *K pneumoniae* in the causal chain, the duration of admission was long enough (>48 h) for *K pneumoniae* to have been acquired during hospitalisation. The epidemiology of *K pneumoniae* as a nosocomial pathogen is well characterised, and nosocomial outbreaks of *K pneumoniae* in neonatal intensive care contribute importantly to hospital-acquired *K pneumoniae* infections in children.^1^^,^^2^ During the study period, documented *K pneumoniae* outbreaks occurred in the neonatal intensive care wards at CHAMPS enrolment sites in South Africa^23^ and Sierra Leone. However, 42% of overall in-hospital deaths with *K pneumoniae* in the causal chain occurred within 48 h of admission, suggesting that *K pneumoniae* might have been acquired before admission. We also observed deaths with *K pneumoniae* in the causal chain that occurred in the community among children with limited or no contact with health-care facilities during the illness that led to their death. Data on community-acquired *K pneumoniae* infections in children are sparse.^24^^,^^25^ Reducing deaths due to *K pneumoniae* will require a better understanding of sources of infection in the community as well as risk factors for developing *K pneumoniae* disease given colonisation.^26^

Distinguishing the exact role of *K pneumoniae* in a child’s death based on post-mortem specimens is difficult because *K pneumoniae* commonly colonises the gastrointestinal tract and nasopharynx,^5^ so detection of the pathogen postmortem could potentially reflect agonal spread, post-mortem translocation, post-mortem overgrowth, or contamination. Detection of *K pneumoniae* was common among deaths in CHAMPS during the study period, with over half of all deaths having *K pneumoniae* detected by TAC on at least one specimen (including nasopharyngeal swabs); because *K pneumoniae* was not tested for in the stool, this likely is an underestimate of the frequency of *K pneumoniae* colonisation among child deaths. However, the comprehensive testing in CHAMPS, which includes microbiology, molecular assays, histopathology, and IHC linked to clinical presentation, provides important insight into the pathogenic role of *K pneumoniae*. Histopathology and IHC aid in distinguishing between post-mortem contamination and antemortem infection because the pathogen can be observed in affected tissues. Also, CHAMPS MITS collection methods reduce the risk of contamination and post-mortem translocation by using sterile procedures and minimising the time between death and sample collection.^27^^,^^28^ Among cases deemed to have *K pneumoniae* in the causal pathway, 68% had *K pneumoniae* detected by both culture and TAC from normally sterile specimens, and many had additional supporting evidence from IHC. Nonetheless, it is possible that some *K pneumoniae* identified post mortem could reflect invasion at the time of dying rather than the true cause of death. Surveillance for severe disease with extensive laboratory testing conducted at the CHAMPS sites could provide complementary data to help better characterise the role of *K pneumoniae* in child illness and death by examining the burden of *K pneumoniae* disease among living children and the role of *K pneumoniae* in child death in the same settings.

Among the *K pneumoniae* isolates, resistance was very common to antibiotics recommended by WHO for the treatment of paediatric pneumonia and sepsis, including gentamicin (first-line recommendation together with ampicillin, which is not clinically active against *Klebsiella* spp) and ceftriaxone (second-line recommendation).^29^ Furthermore, among isolates tested for carbapenem resistance (imipenem and meropenem), 21–30% were resistant, and carbapenem-resistant *K pneumoniae* infections are associated with increased mortality, based on data from primarily high-income settings.^11^^,^^12^ Risk factors for carbapenem-resistant *K pneumoniae* infections have been described for predominantly adult populations,^30^ but data for children are limited. The findings of our study highlight the need for clinical predictors of *K pneumoniae* infections and rapid diagnostics in order to identify children who need antibiotics that would cover *K pneumoniae*, as well as monitoring of *K pneumoniae* resistance patterns. Furthermore, guidelines for empirical treatment of pneumonia and sepsis might require adaptation to optimally prevent deaths due to *K pneumoniae*. Infection prevention and control practices and antimicrobial stewardship efforts are also important for curbing the spread of resistant *K pneumoniae* strains.

The non-standardisation of antimicrobial susceptibility testing limits the interpretation and generalisability of the antimicrobial resistance findings. We did not perform genomic characterisation, so it is unknown whether any of the deaths with *K pneumoniae* in the causal pathway involved the emerging hypervirulent pathotype;^8^ however, this analysis is planned. Small numbers from several sites limited our ability to compare and contrast across sites. As noted above, it is possible that for some deaths found to have *K pneumoniae* in the causal chain, *K pneumoniae* infection represented agonal spread or contamination. Cases with less robust evidence of *K pneumoniae* involvement might have been misclassified as having *K pneumoniae* in the causal chain, potentially leading to an overestimation of the role of *K pneumoniae* in child deaths. Despite efforts to comprehensively capture all eligible deaths in the CHAMPS catchment areas, the sample of deaths included in this analysis might not be entirely representative of all deaths occurring in the surveillance area.

We present evidence that *K pneumoniae* substantially contributed to community and facility deaths in the first 2 years of life across multiple high-mortality settings. Further research is needed to better understand the source of infection and risk factors for *K pneumoniae* mortality. Given the burden of *K pneumoniae* and the frequent resistance to first-line antibiotics used for pneumonia, sepsis, and meningitis, improved strategies are needed to rapidly identify children who might be infected with *K pneumoniae* and need more appropriate antibiotic treatment. Our data also suggest a potential impact of developing and using effective *K pneumoniae* vaccines in reducing neonatal, infant, and child deaths globally.^31^

## Data sharing

CHAMPS data are available online at https://champshealth.org/.

## Declaration of interests

SEA, KLK, and MT report receiving funding from Emory University. JAGS reports receiving funding from Emory University, the Bill & Melinda Gates Foundation, Wellcome Trust, UK Foreign Commonwealth & Development Office, European Union, and National Institute for Health Research. SMad reports receiving funds from the Bill & Melinda Gates Foundation, Pfizer, Minervax, GSK, South African Medical Research Council, Merck, PATH, and Center for the AIDS Programme of Research in South Africa. All other authors declare no competing interests.
