# Supplementary material for: Child deaths caused by Klebsiella pneumoniae in sub-Saharan Africa and south Asia: a secondary analysis of Child Health and Mortality Prevention Surveillance (CHAMPS) data
Source: Lancet Microbe. 2024 Feb;5(2):e131–41. doi: 10.1016/S2666-5247(23)00290-2 (PMC10849973; doi:10.1016/S2666-5247(23)00290-2)
Supplement: Supplementary appendix [file mmc1.pdf]

THE LANCET
Microbe

Supplementary appendix

This appendix formed part of the original submission and has been peer reviewed.
We post it as supplied by the authors.

Supplement to: Verani JR, Blau DM, Gurley ES, et al. Child deaths caused by *Klebsiella pneumoniae* in sub-Saharan Africa and south Asia: a secondary analysis of Child Health and Mortality Prevention Surveillance (CHAMPS) data. *Lancet Microbe* 2024. https://doi.org/10.1016/S2666-5247(23)00290-2

Supplemental Material

Table of Contents

Figure S1 Flowchart of enrolled stillbirths and under-5 deaths from CHAMPS sites between December 2016 – December 2021, that had minimally invasive tissue samples (MITS) and consent only for verbal autopsy and clinical abstraction (Non-MITS) and included in the analysis

Table S1 Pathogens involved in multi-pathogen sepsis or pneumonia with *Klebsiella pneumoniae*

Table S2 Underlying causes of death among deaths with Kp as immediate cause of death or morbid condition among all sites, by age group

Table S3 Underlying causes of death among deaths with Kp as immediate cause of death or morbid condition among all sites, by site

Table S4 Factors associated with *Klebsiella pneumoniae* in the causal chain, CHAMPS, 2016-2021

Figure S1: Flowchart of enrolled stillbirths and under-5 deaths from CHAMPS sites between December 2016 – December 2021, that had minimally invasive tissue samples (MITS) and consent only for verbal autopsy and clinical abstraction (Non-MITS) and included in the analysis

a. Stillbirths

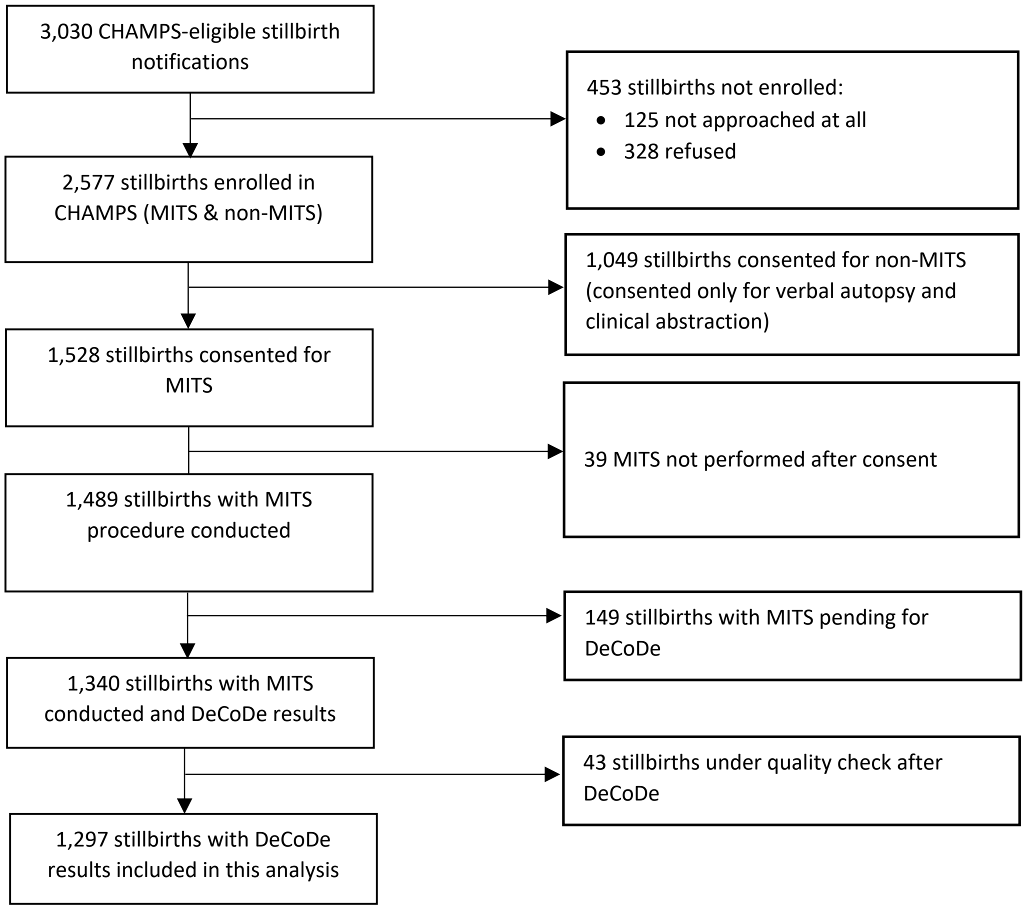

b. Child deaths

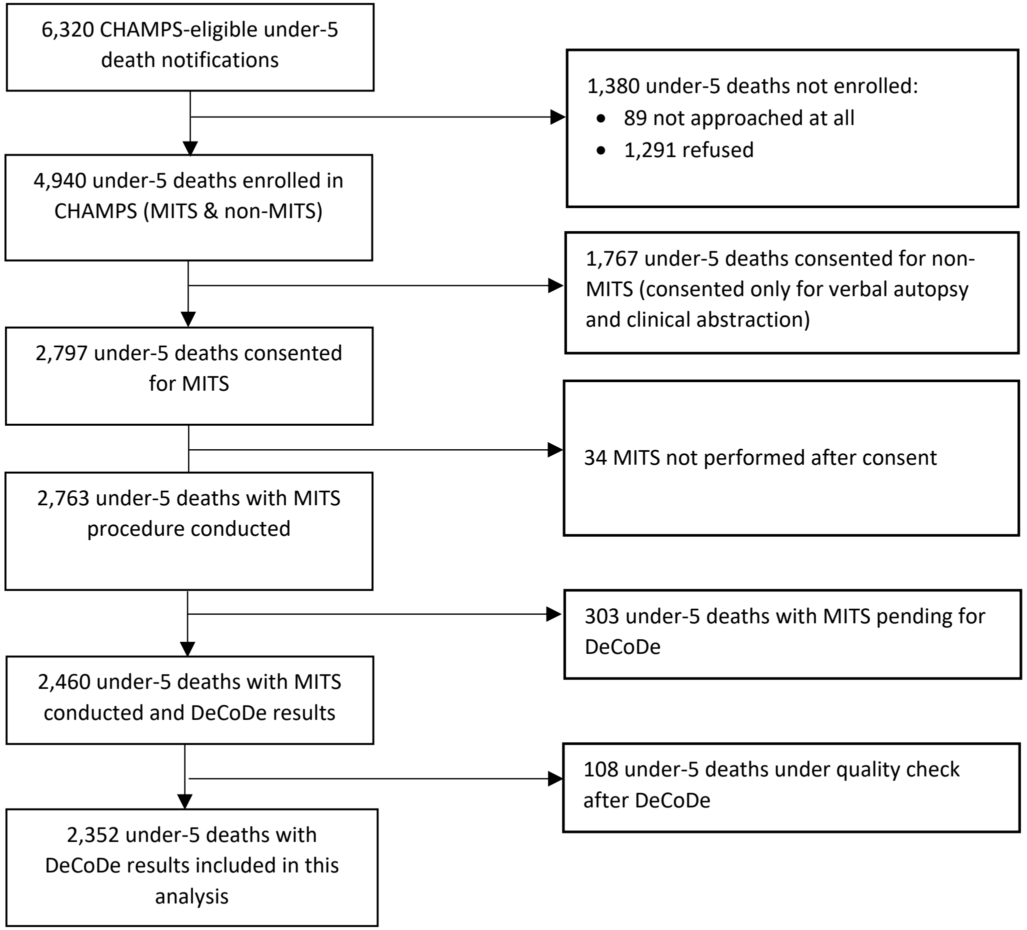

Table S1. Pathogens involved in multi-pathogen sepsis or pneumonia with *Klebsiella pneumoniae*								
Pathogen	Community (n=29)		Hospital death ≤48 hours after admission (n=91)		Hospital death >48 hours after admission (n=153)		Total (n=273)	
	n	% (95% CI)	n	% (95% CI)	n	% (95% CI)	n	% (95% CI)
Acinetobacter baumannii	1	3 (0, 20)	10	11 (6, 20)	76	50 (42, 58)	87	32 (26, 38)
Acinetobacter spp.	0	0 (0, 15)	1	1 (0, 7)	0	0 (0, 3)	1	0 (0, 2)
Adenovirus	0	0 (0, 15)	0	0 (0, 5)	1	1 (0, 4)	1	0 (0, 2)
Bordetella spp.	0	0 (0, 15)	1	1 (0, 7)	0	0 (0, 3)	1	0 (0, 2)
Brevundimonas vesicularis	0	0 (0, 15)	0	0 (0, 5)	1	1 (0, 4)	1	0 (0, 2)
Candida albicans	2	7 (1, 24)	4	4 (1, 11)	6	4 (2, 9)	12	4 (2, 8)
Candida auris	0	0 (0, 15)	0	0 (0, 5)	1	1 (0, 4)	1	0 (0, 2)
Candida glabrata	0	0 (0, 15)	0	0 (0, 5)	1	1 (0, 4)	1	0 (0, 2)
Candida spp.	0	0 (0, 15)	1	1 (0, 7)	4	3 (1, 7)	5	2 (1, 4)
Cytomegalovirus	3	10 (3, 28)	7	8 (3, 16)	6	4 (2, 9)	16	6 (3, 10)
Enterobacter spp.	0	0 (0, 15)	1	1 (0, 7)	0	0 (0, 3)	1	0 (0, 2)
Enterobacter cloacae	2	7 (1, 24)	4	4 (1, 11)	4	3 (1, 7)	10	4 (2, 7)
Enterococcus faecalis	1	3 (0, 20)	5	5 (2, 13)	9	6 (3, 11)	15	5 (3, 9)
Enterococcus faecium	0	0 (0, 15)	3	3 (1, 10)	7	5 (2, 10)	10	4 (2, 7)
Enterococcus spp.	0	0 (0, 15)	1	1 (0, 7)	3	2 (1, 6)	4	1 (0, 4)
Enterovirus	0	0 (0, 15)	0	0 (0, 5)	1	1 (0, 4)	1	0 (0, 2)
Escherichia coli	9	31 (16, 51)	24	26 (18, 37)	24	16 (11, 23)	57	21 (16, 26)
Escherichia coli/Shigella spp.	0	0 (0, 15)	3	3 (1, 10)	5	3 (1, 8)	8	3 (1, 6)
Group A Streptococcus	0	0 (0, 15)	1	1 (0, 7)	0	0 (0, 3)	1	0 (0, 2)
Haemophilus influenzae	3	10 (3, 28)	6	7 (3, 14)	4	3 (1, 7)	13	5 (3, 8)
Haemophilus influenzae Type A	1	3 (0, 20)	2	2 (0, 8)	2	1 (0, 5)	5	2 (1, 4)
Haemophilus influenzae Type B	0	0 (0, 15)	1	1 (0, 7)	0	0 (0, 3)	1	0 (0, 2)
Human coronavirus OC43	0	0 (0, 15)	0	0 (0, 5)	1	1 (0, 4)	1	0 (0, 2)
Human metapneumovirus	0	0 (0, 15)	1	1 (0, 7)	3	2 (1, 6)	4	1 (0, 4)
Influenza A	0	0 (0, 15)	1	1 (0, 7)	1	1 (0, 4)	2	1 (0, 3)
Influenza B	0	0 (0, 15)	1	1 (0, 7)	0	0 (0, 3)	1	0 (0, 2)
Klebsiella oxytoca	0	0 (0, 15)	1	1 (0, 7)	1	1 (0, 4)	2	1 (0, 3)
Klebsiella terragina	0	0 (0, 15)	0	0 (0, 5)	1	1 (0, 4)	1	0 (0, 2)
Moraxella catarrhalis	0	0 (0, 15)	1	1 (0, 7)	0	0 (0, 3)	1	0 (0, 2)
Morganella morgani	0	0 (0, 15)	1	1 (0, 7)	0	0 (0, 3)	1	0 (0, 2)
Parainfluenza virus type 3	0	0 (0, 15)	1	1 (0, 7)	2	1 (0, 5)	3	1 (0, 3)
Parainfluenza virus type 4	1	3 (0, 20)	0	0 (0, 5)	0	0 (0, 3)	1	0 (0, 2)
Pneumocystis jirovecii	4	14 (5, 33)	5	5 (2, 13)	2	1 (0, 5)	11	4 (2, 7)
Proteus mirabilis	0	0 (0, 15)	0	0 (0, 5)	1	1 (0, 4)	1	0 (0, 2)
Proteus vulgaris	0	0 (0, 15)	1	1 (0, 7)	0	0 (0, 3)	1	0 (0, 2)
Pseudomonas aeruginosa	3	10 (3, 28)	12	13 (7, 22)	15	10 (6, 16)	30	11 (8, 15)
Respiratory syncytial virus	0	0 (0, 15)	5	5 (2, 13)	5	3 (1, 8)	10	4 (2, 7)
Rhinovirus	0	0 (0, 15)	0	0 (0, 5)	2	1 (0, 5)	2	1 (0, 3)
Salmonella spp.	0	0 (0, 15)	3	3 (1, 10)	5	3 (1, 8)	8	3 (1, 6)
Serratia marcescens	0	0 (0, 15)	0	0 (0, 5)	1	1 (0, 4)	2	1 (0, 3)
Shigella	0	0 (0, 15)	1	1 (0, 7)	0	0 (0, 3)	1	0 (0, 2)
Staphylococcus aureus	3	10 (3, 28)	5	5 (2, 13)	10	7 (3, 12)	18	7 (4, 10)
Staphylococcus epidermidis	0	0 (0, 15)	0	0 (0, 5)	1	1 (0, 4)	1	0 (0, 2)
Streptococcus agalactiae	0	0 (0, 15)	1	1 (0, 7)	4	3 (1, 7)	5	2 (1, 4)
Streptococcus Group G	0	0 (0, 15)	1	1 (0, 7)	0	0 (0, 3)	1	0 (0, 2)
Streptococcus pneumoniae	13	45 (27, 64)	26	29 (20, 39)	6	4 (2, 9)	45	16 (12, 22)
Streptococcus pyogenes	1	3 (0, 20)	1	1 (0, 7)	2	1 (0, 5)	4	1 (0, 4)
Streptococcus spp.	3	10 (3, 28)	7	8 (3, 16)	5	3 (1, 8)	15	5 (3, 9)
Streptococcus viridans	0	0 (0, 15)	0	0 (0, 5)	1	1 (0, 4)	1	0 (0, 2)
Ureaplasma spp.	0	0 (0, 15)	1	1 (0, 7)	1	1 (0, 4)	2	1 (0, 3)

Table S2. Underlying causes of death among deaths with Kp as immediate cause of death or morbid condition among all sites, by age group								
	Neonate (n=195)		1-11 months (n=116)		12-59 months (n=86)		Total (n=397)	
Underlying cause of death	n	% (95% CI)	n	% (95% CI)	n	% (95% CI)	n	% (95% CI)
Neonatal preterm birth complications	109	56 (49, 63)	24	21 (14, 29)	0	0 (0, 5)	133	34 (29, 38)
Malnutrition	0	0 (0, 2)	32	28 (20, 37)	30	35 (25, 46)	62	16 (12, 20)
Congenital birth defects	24	12 (8, 18)	20	17 (11, 26)	4	5 (2, 12)	48	12 (9, 16)
Perinatal asphyxia/hypoxia	40	21 (15, 27)	1	1 (0, 5)	0	0 (0, 5)	41	10 (8, 14)
HIV	0	0 (0, 2)	13	11 (6, 19)	14	16 (10, 26)	27	7 (5, 10)
Diarrheal Diseases	1	1 (0, 3)	8	7 (3, 14)	8	9 (4, 18)	17	4 (3, 7)
Injury	0	0 (0, 2)	1	1 (0, 5)	14	16 (10, 26)	15	4 (2, 6)
Congenital infection	4	2 (1, 6)	3	3 (1, 8)	1	1 (0, 7)	8	2 (1, 4)
Malaria	0	0 (0, 2)	0	0 (0, 4)	7	8 (4, 17)	7	2 (1, 4)
Lower respiratory infections	1	1 (0, 3)	4	3 (1, 9)	0	0 (0, 5)	5	1 (0, 3)
Neonatal aspiration syndromes	5	3 (1, 6)	0	0 (0, 4)	0	0 (0, 5)	5	1 (0, 3)
Other neonatal disorders	3	2 (0, 5)	1	1 (0, 5)	0	0 (0, 5)	4	1 (0, 3)
Other neurological disorders	0	0 (0, 2)	2	2 (0, 7)	2	2 (0, 9)	4	1 (0, 3)
Sepsis	3	2 (0, 5)	1	1 (0, 5)	0	0 (0, 5)	4	1 (0, 3)
Neonatal encephalopathy	2	1 (0, 4)	1	1 (0, 5)	0	0 (0, 5)	3	1 (0, 2)
Syphilis	1	1 (0, 3)	2	2 (0, 7)	0	0 (0, 5)	3	1 (0, 2)
Liver disease	0	0 (0, 2)	0	0 (0, 4)	2	2 (0, 9)	2	1 (0, 2)
Measles	0	0 (0, 2)	0	0 (0, 4)	2	2 (0, 9)	2	1 (0, 2)
Other	1	1 (0, 3)	1	1 (0, 5)	0	0 (0, 5)	2	1 (0, 2)
Birth trauma	1	1 (0, 3)	0	0 (0, 4)	0	0 (0, 5)	1	0 (0, 2)
Cancer	0	0 (0, 2)	0	0 (0, 4)	1	1 (0, 7)	1	0 (0, 2)
Other infections	0	0 (0, 2)	1	1 (0, 5)	0	0 (0, 5)	1	0 (0, 2)
Other respiratory disease	0	0 (0, 2)	0	0 (0, 4)	1	1 (0, 7)	1	0 (0, 2)
Other skin and subcutaneous diseases	0	0 (0, 2)	1	1 (0, 5)	0	0 (0, 5)	1	0 (0, 2)

Table S3. Underlying causes of death among deaths with Kp as immediate cause of death or morbid condition among all sites, by site																
	South Africa (n=167)		Kenya (n=33)		Mozambique (n=35)		Sierra Leone (n=73)		Bangladesh (n=7)		Mali (n=19)		Ethiopia (n=63)		Total (n=397)	
Underlying cause of death	n	% (95% CI)	n	% (95% CI)	n	% (95% CI)	n	% (95% CI)	n	% (95% CI)	n	% (95% CI)	n	% (95% CI)	n	% (95% CI)
Neonatal preterm birth complications	79	47 (40, 55)	6	18 (8, 36)	5	14 (5, 31)	6	8 (3, 18)	5 (71)	71 (30, 95)	6	32 (14, 57)	26	41 (29, 54)	133	34 (29, 38)
Malnutrition	5	3 (1, 7)	13	39 (23, 58)	4	11 (4, 28)	29	40 (29, 52)	1 (14)	14 (1, 58)	1	5 (0, 28)	9	14 (7, 26)	62	16 (12, 20)
Congenital birth defects	32	19 (14, 26)	5	15 (6, 33)	4	11 (4, 28)	0	0 (0, 6)	0 (0)	0 (0, 44)	2	11 (2, 35)	5	8 (3, 18)	48	12 (9, 16)
Perinatal asphyxia/hypoxia	4	2 (1, 6)	0	0 (0, 13)	2	6 (1, 21)	19	26 (17, 38)	1 (14)	14 (1, 58)	1	5 (0, 28)	14	22 (13, 35)	41	10 (8, 14)
HIV	10	6 (3, 11)	1	3 (0, 18)	8	23 (11, 41)	6	8 (3, 18)	0 (0)	0 (0, 44)	2	11 (2, 35)	0	0 (0, 7)	27	7 (5, 10)
Diarrheal Diseases	5	3 (1, 7)	0	0 (0, 13)	10	29 (15, 47)	2	3 (0, 10)	0 (0)	0 (0, 44)	0	0 (0, 21)	0	0 (0, 7)	17	4 (3, 7)
Injury	9	5 (3, 10)	2	6 (1, 22)	0	0 (0, 12)	3	4 (1, 12)	0 (0)	0 (0, 44)	1	5 (0, 28)	0	0 (0, 7)	15	4 (2, 6)
Congenital infection	6	4 (1, 8)	1	3 (0, 18)	0	0 (0, 12)	0	0 (0, 6)	0 (0)	0 (0, 44)	1	5 (0, 28)	0	0 (0, 7)	8	2 (1, 4)
Malaria	0	0 (0, 3)	0	0 (0, 13)	1	3 (0, 17)	6	8 (3, 18)	0 (0)	0 (0, 44)	0	0 (0, 21)	0	0 (0, 7)	7	2 (1, 4)
Lower respiratory infections	2	1 (0, 5)	1	3 (0, 18)	0	0 (0, 12)	1	1 (0, 8)	0 (0)	0 (0, 44)	0	0 (0, 21)	1	2 (0, 10)	5	1 (0, 3)
Neonatal aspiration syndromes	0	0 (0, 3)	0	0 (0, 13)	0	0 (0, 12)	0	0 (0, 6)	0 (0)	0 (0, 44)	0	0 (0, 21)	5	8 (3, 18)	5	1 (0, 3)
Other neonatal disorders	2	1 (0, 5)	1	3 (0, 18)	0	0 (0, 12)	0	0 (0, 6)	0 (0)	0 (0, 44)	1	5 (0, 28)	0	0 (0, 7)	4	1 (0, 3)
Other neurological disorders	2	1 (0, 5)	1	3 (0, 18)	0	0 (0, 12)	0	0 (0, 6)	0 (0)	0 (0, 44)	1	5 (0, 28)	0	0 (0, 7)	4	1 (0, 3)
Sepsis	4	2 (1, 6)	0	0 (0, 13)	0	0 (0, 12)	0	0 (0, 6)	0 (0)	0 (0, 44)	0	0 (0, 21)	0	0 (0, 7)	4	1 (0, 3)
Neonatal encephalopathy	1	1 (0, 4)	0	0 (0, 13)	1	3 (0, 17)	0	0 (0, 6)	0 (0)	0 (0, 44)	0	0 (0, 21)	1	2 (0, 10)	3	1 (0, 2)
Syphilis	3	2 (0, 6)	0	0 (0, 13)	0	0 (0, 12)	0	0 (0, 6)	0 (0)	0 (0, 44)	0	0 (0, 21)	0	0 (0, 7)	3	1 (0, 2)
Liver disease	1	1 (0, 4)	0	0 (0, 13)	0	0 (0, 12)	0	0 (0, 6)	0 (0)	0 (0, 44)	0	0 (0, 21)	1	2 (0, 10)	2	1 (0, 2)
Measles	0	0 (0, 3)	0	0 (0, 13)	0	0 (0, 12)	0	0 (0, 6)	0 (0)	0 (0, 44)	1	5 (0, 28)	1	2 (0, 10)	2	1 (0, 2)
Other	0	0 (0, 3)	0	0 (0, 13)	0	0 (0, 12)	1	1 (0, 8)	0 (0)	0 (0, 44)	1	5 (0, 28)	0	0 (0, 7)	2	1 (0, 2)
Birth trauma	0	0 (0, 3)	0	0 (0, 13)	0	0 (0, 12)	0	0 (0, 6)	0 (0)	0 (0, 44)	1	5 (0, 28)	0	0 (0, 7)	1	0 (0, 2)
Cancer	1	1 (0, 4)	0	0 (0, 13)	0	0 (0, 12)	0	0 (0, 6)	0 (0)	0 (0, 44)	0	0 (0, 21)	0	0 (0, 7)	1	0 (0, 2)
Other infections	1	1 (0, 4)	0	0 (0, 13)	0	0 (0, 12)	0	0 (0, 6)	0 (0)	0 (0, 44)	0	0 (0, 21)	0	0 (0, 7)	1	0 (0, 2)
Other respiratory disease	0	0 (0, 3)	1	3 (0, 18)	0	0 (0, 12)	0	0 (0, 6)	0 (0)	0 (0, 44)	0	0 (0, 21)	0	0 (0, 7)	1	0 (0, 2)
Other skin and subcutaneous diseases	0	0 (0, 3)	1	3 (0, 18)	0	0 (0, 12)	0	0 (0, 6)	0 (0)	0 (0, 44)	0	0 (0, 21)	0	0 (0, 7)	1	0 (0, 2)

**Table S4**. Factors associated with *Klebsiella pneumoniae* in the causal chain, CHAMPS, 2016-2021						
	**Kp in causal chain, n (%) n = 497**	**Kp not in causal chain, n (%) n = 1855**	**Odds Ratio (95% CI)**	***P* value**	**Adjusted* Odds Ratio (95% CI)**	***P* value**
**Age at the time of death**				<0.001		<0.001
Neonate	257 (52)	1167 (63)	*Referent*		*Referent*	
1-11 months	144 (29)	341 (18)	1.92 (1.51, 2.43)		2.26 (1.72, 2.96)	
12-23 months	63 (13)	162 (9)	1.77 (1.27, 2.42)		2.02 (1.40, 2.89)	
24-59 months	33 (7)	185 (10)	0.81 (0.54, 1.19)		0.94 (0.60, 1.43)	
**Location of death**				<0.001		<0.001
Facility ≤48 hours	188 (38)	1142 (62)	*Referent*		*Referent*	
Facility >48 hours	262 (53)	431 (23)	3.69 (2.97, 4.60)		3.98 (3.11, 5.12)	
Community	47 (9)	282 (15)	1.01 (0.71, 1.42)		0.81 (0.55, 1.18)	
**Site**				<0.001		<0.001
Bangladesh	11 (2)	173 (9)	0.18 (0.09, 0.33)		0.37 (0.18, 0.68)	
Ethiopia	71 (14)	65 (4)	3.15 (2.16, 4.60)		6.70 (4.42, 10.21)	
Kenya	51 (10)	381 (21)	0.39 (0.27, 0.54)		0.61 (0.41, 0.88)	
Mali	21 (4)	105 (6)	0.58 (0.34, 0.93)		1.36 (0.78, 2.29)	
Mozambique	55 (11)	373 (20)	0.43 (0.30, 0.59)		0.61 (0.43, 0.87)	
Sierra Leone	107 (22)	236 (13)	1.31 (0.98, 1.74)		1.90 (1.38, 2.63)	
South Africa	181 (36)	522 (28)	*Referent*		*Referent*	
*Adjusted for all other variables listed in the model						
